# Influence of the concentration of dietary digestible calcium on growth performance, bone mineralization, plasma calcium, and abundance of genes involved in intestinal absorption of calcium in pigs from 11 to 22 kg fed diets with different concentrations of digestible phosphorus

**DOI:** 10.1186/s40104-019-0349-2

**Published:** 2019-05-28

**Authors:** L. Vanessa Lagos, Su A. Lee, Guillermo Fondevila, Carrie L. Walk, Michael R. Murphy, Juan J. Loor, Hans H. Stein

**Affiliations:** 1 Division of Nutritional Sciences, University of Illinois, Urbana, IL 61801 USA; 2 Department of Animal Sciences, University of Illinois, Urbana, IL 61801 USA; 3 Universidad Politécnica de Madrid, Madrid, Spain; 4 AB Vista, Marlborough, SN8 4AN UK

**Keywords:** Bone ash, Calcium absorption, Digestible calcium, Growth, Pigs, Requirement

## Abstract

**Background:**

A 21-day experiment was conducted to test the hypothesis that Ca requirements to maximize growth performance expressed as the standardized total tract digestible (STTD) Ca to STTD P ratio is less than 1.40:1. The second hypothesis was that increasing dietary Ca increases plasma Ca concentration and downregulates abundance of genes related to Ca absorption (*TRPV6*, *S100G*, and *ATP2B1*) in the duodenum, and tight junction proteins (*OCLN*, *CLDN1*, and *ZO1*) in the duodenum and ileum.

**Methods:**

Twenty corn-soybean meal diets were formulated using a 4 × 5 factorial design with diets containing 0.16%, 0.33%, 0.42%, or 0.50% STTD P, and 0.14%, 0.29%, 0.44%, 0.59%, or 0.74% STTD Ca. Six hundred and forty pigs (initial weight: 11.1 ± 1.4 kg) were allotted to 20 diets and 5 blocks in a randomized complete block design. On day 21, weights of pigs and feed left in feeders were recorded and blood, duodenal tissue, ileal mucosa, and the right femur were collected from 1 pig per pen. Abundance of mRNA was determined in duodenal and ileal tissue via quantitative RT-PCR. Data were analyzed using a response surface model.

**Results:**

The predicted maximum ADG (614 g), G:F (0.65), and bone ash (11.68 g) was obtained at STTD Ca:STTD P ratios of 1.39:1, 1.25:1, and 1.66:1, respectively, when STTD P was provided at the requirement (0.33%). If dietary STTD P was below the requirement, increasing dietary Ca resulted in reduced (*P* < 0.05) ADG and G:F. However, if dietary STTD P was above the requirement, negative effects (*P* < 0.05) on ADG and G:F of increasing STTD Ca were observed only if dietary STTD Ca exceeded 0.6%. Plasma Ca concentration was positively affected by STTD Ca over the range studied (quadratic, *P* < 0.01) and negatively affected by increasing STTD P (linear, *P* < 0.01). There was a linear negative effect (*P* < 0.05) of STTD Ca on the abundance of *S100G*, *TRPV6*, *OCLN*, and *ZO1* in duodenum, and *CLDN* and *ZO1* in ileum.

**Conclusions:**

The STTD Ca:STTD P ratio needed to maximize growth performance of 11- to 25-kg pigs is less than 1.40:1, if P is at the estimated requirement. Increasing dietary Ca reduces transcellular absorption of Ca and increases paracellular absorption of Ca.

**Electronic supplementary material:**

The online version of this article (10.1186/s40104-019-0349-2) contains supplementary material, which is available to authorized users.

## Background

Requirements for P have been expressed as standardized total tract digestible (STTD) P, and although it is believed that diets for pigs will be more accurately formulated based on a STTD Ca:STTD P ratio, Ca requirements have been expressed as total Ca because of a lack of data for the digestibility of Ca in feed ingredients [1]. However, recent work has generated values for STTD of Ca in most Ca containing feed ingredients [2], which has allowed for formulating diets for growing pigs based on STTD Ca. It has also been demonstrated that a ratio between STTD Ca and STTD P that is less than 1.35:1, 1.25:1, and 1.10:1 maximizes growth performance of pigs from 25 to 50 kg [3], 50 to 85 kg [4], and 100 to 130 kg [5], respectively, if STTD P is provided at the requirement [1]. It is therefore possible that pigs from 11 to 25 kg require a ratio between STTD Ca and STTD P that may be as high as 1.40:1. It was also reported that the STTD Ca:STTD P ratio needed to maximize bone ash is greater than the ratio needed to maximize growth performance [3–5]. An attempt to estimate the requirement for STTD Ca by pigs from 11 to 25 kg was also made, but due to a reduction in ADG and G:F as dietary Ca increased, an optimal STTD Ca:STTD P ratio could not be estimated [6]. It was therefore concluded from this work that to obtain requirements for STTD Ca in pigs from 11 to 25 kg, it is necessary to not only use increasing dietary concentrations of STTD Ca, but also to use diets with different concentrations of dietary P.

Calcium is absorbed by transcellular or paracellular transport [7]. Transcellular transport is the primary route if dietary Ca is low and this absorption is stimulated by vitamin D [8]. In contrast, if dietary Ca is adequate or high, increased quantities of Ca are absorbed using paracellular absorption via the tight junctions [9]. Data for effects of dietary Ca on mRNA abundance of transcellular transporters for Ca in the jejunum and kidneys of pigs were reported [6], but there are limited data demonstrating effects of dietary Ca on abundance of genes related to paracellular transport of Ca in pigs. Therefore, the objectives of this experiment were to test the hypotheses that a STTD Ca:STTD P ratio less than 1.40:1 maximizes growth performance of pigs from 11 to 25 kg and that increasing dietary Ca increases plasma concentration of Ca and downregulates abundance of genes related to transcellular absorption and transport of Ca in the duodenum and tight junction proteins in the duodenum and ileum.

## Methods

The Institutional Animal Care and Use Committee at the University of Illinois reviewed and approved the protocol for the experiment. Pigs used in the experiment were the offspring of Line 359 boars and Camborough females (Pig Improvement Company, Hendersonville, TN).

### Animals and housing

Six hundred and forty pigs with an initial average body weight (BW) of 11.1 ± 1.4 kg were randomly allotted to 20 diets and 5 blocks in a randomized complete bock design. The average pen weights were 11.1 ± 1.2 kg. Pigs were blocked based on weaning date and BW within weaning date. Blocks 1, 4, and 5 had 2 replicate pens per diet (40 pens) and blocks 2 and 3 had 1 replicate pen per diet (20 pens). Therefore, there were 8 replicate pens per diet in total. There were 2 barrows and 2 gilts in each pen (1.2 m × 1.4 m) and pens had fully slatted floors, a feeder, and a nipple drinker. Rooms were ventilated using a negative pressure ventilation system and heat was applied to maintain desired barn temperatures. Feed and water were available at all times. The Experimental Animal Allotment Program [10] was used to allot pigs to experimental diets based on BW and sex.

### Diets and feeding

Twenty corn-soybean meal-based diets were formulated to have different concentrations of Ca and P, but the amount of corn and soybean meal was constant among diets to keep the concentration of phytate constant (Table 1). Diets were mixed in a 1000-kg ribbon mixer and stored in bags until used. One batch of each diet was mixed. Diets were formulated using a 4 × 5 factorial design to contain 0.16%, 0.33%, 0.42%, or 0.50% STTD P, and 0.21%, 0.45%, 0.70%, 0.94%, or 1.19% total Ca, which correspond to 0.14%, 0.29%, 0.44%, 0.59%, and 0.74% STTD Ca, respectively (Tables 2 and 3). Diets were formulated using values for total Ca and P from NRC [1] and values for STTD P and STTD Ca of ingredients from NRC [1] and Stein et al. [2], respectively. The four levels of dietary P corresponded to 50%, 100%, 127%, and 151% of the estimated requirement of 0.33% STTD P [1]. The five levels of dietary Ca corresponded to 30%, 64%, 100%, 134%, and 170% of the estimated requirement for total Ca [1]. The concentration of P was increased by increasing the monosodium phosphate inclusion, but to maintain a constant concentration of Na among diets, inclusion of sodium bicarbonate was reduced as the concentration of monosodium phosphate increased.

**Table 1:** Analyzed composition of ingredients used in diets, as fed basis

Item	Corn	Soybean meal	Lactose	Calcium carbonate	Monocalcium phosphate	Monosodium phosphate	Sodium bicarbonate	Sodium chloride
GE, kcal/kg	3769	4173	3694	–	–	–	–	–
DM, %	85.29	86.97	94.91	99.94	92.47	99.83	63.33	99.96
Ash, %	1.49	7.11	0.19	91.53	81.89	91.85	63.30	100.18
CP, %	6.43	46.60	–	–	–	–	–	–
AEE^a^, %	4.31	1.45	–	–	–	–	–	–
ADF, %	3.02	6.60	–	–	–	–	–	–
NDF, %	9.16	7.08	–	–	–	–	–	–
Ca, %	0.02	0.30	0.02	39.23	18.59	0.04	0.01	0.19
P, %	0.29	0.67	0.01	0.02	21.59	25.44	0.03	ND^b^
Phytate^c^, %	0.74	1.49	–	–	–	–	–	–
Phytate-bound P, %	0.21	0.42	–	–	–	–	–	–
Non-phytate P^d^, %	0.08	0.25	–	–	–	–	–	–
Na, %	0.02	0.02	–	0.05	0.09	19.04	27.10	39.45
Cl, %	0.10	0.10	–	ND	0.01	ND	ND	58.77
K, %	0.44	2.49	–	0.10	0.16	0.22	ND	ND

^a^AEE acid hydrolyzed ether extract

^b^ND not detectable

^c^Phytate was calculated by dividing the phytate-bound P by 0.282 (Tran and Sauvant [16])

^d^Non-phytate P was calculated as the difference between total P and phytate-bound P

**Table 2:** Ingredient composition and analyzed composition of experimental diets containing 0.16% and 0.33% standardized total tract digestible (STTD) P, as-fed basis^a^

Ingredient, %	0.16% STTD P					0.33% STTD P				
Total P, %:	0.35	0.35	0.35	0.35	0.35	0.53	0.53	0.53	0.53	0.53
Total Ca, %:	0.21	0.45	0.70	0.95	1.19	0.21	0.45	0.70	0.95	1.19
STTD Ca, %:	0.14	0.29	0.44	0.59	0.74	0.14	0.29	0.44	0.59	0.74
Corn	46.00	46.00	46.00	46.00	46.00	46.00	46.00	46.00	46.00	46.00
Soybean meal	32.50	32.50	32.50	32.50	32.50	32.50	32.50	32.50	32.50	32.50
Cornstarch	8.85	7.05	5.11	3.26	1.43	8.39	6.59	4.71	2.81	0.97
Lactose	10.00	10.00	10.00	10.00	10.00	10.00	10.00	10.00	10.00	10.00
Choice white grease	–	1.17	2.45	3.65	4.85	0.30	1.47	2.70	3.95	5.15
Calcium carbonate	0.16	0.79	1.45	2.10	2.73	0.16	0.79	1.44	2.10	2.73
Monocalcium phosphate	0.10	0.10	0.10	0.10	0.10	0.10	0.10	0.10	0.10	0.10
Monosodium phosphate	–	–	–	–	–	0.75	0.75	0.75	0.74	0.75
Sodium bicarbonate	1.15	1.15	1.15	1.15	1.15	0.56	0.56	0.56	0.56	0.56
*L*-Lys HCl	0.37	0.37	0.37	0.37	0.37	0.37	0.37	0.37	0.37	0.37
*DL*-Met	0.14	0.14	0.14	0.14	0.14	0.14	0.14	0.14	0.14	0.14
Thr	0.12	0.12	0.12	0.12	0.12	0.12	0.12	0.12	0.12	0.12
Val	0.01	0.01	0.01	0.01	0.01	0.01	0.01	0.01	0.01	0.01
Sodium chloride	0.40	0.40	0.40	0.40	0.40	0.40	0.40	0.40	0.40	0.40
Vitamin mineral premix^b^	0.20	0.20	0.20	0.20	0.20	0.20	0.20	0.20	0.20	0.20
Total	100.0	100.0	100.0	100.0	100.0	100.0	100.0	100.0	100.0	100.0
Analyzed composition										
GE, kcal/kg	3910	3846	3842	3966	3984	3846	3869	3926	3954	3991
DM, %	87.04	87.48	87.70	87.88	88.04	87.92	88.08	88.06	87.24	87.63
CP, %	18.80	19.00	19.23	18.93	19.50	19.42	19.15	18.47	19.17	18.86
Ash, %	3.95	5.03	5.22	6.84	6.73	4.20	4.64	5.55	6.57	6.75
AEE^c^, %	2.67	3.33	5.18	5.94	7.46	3.03	3.60	5.27	6.31	7.56
ADF, %	3.67	4.22	2.84	4.87	3.43	3.25	3.40	3.21	3.23	3.59
NDF, %	6.80	7.26	6.52	7.73	7.00	6.12	6.20	6.41	6.59	6.40
Ca, %	0.26	0.46	0.74	0.93	1.27	0.21	0.46	0.72	1.02	1.24
P, %	0.36	0.36	0.36	0.37	0.37	0.54	0.60	0.58	0.56	0.59
Phytate^d^, %	0.49	0.49	0.49	0.49	0.49	0.49	0.49	0.49	0.49	0.49
Phytate-bound P^e^, %	0.14	0.14	0.14	0.14	0.14	0.14	0.14	0.14	0.14	0.14
Non-phytate P^f^, %	0.22	0.22	0.22	0.23	0.23	0.40	0.46	0.44	0.42	0.45
Na, %	0.46	0.47	0.47	0.49	0.47	0.42	0.50	0.47	0.46	0.47
Cl, %	0.28	0.33	0.32	0.32	0.34	0.33	0.31	0.32	0.33	0.33
K, %	0.92	0.91	0.89	0.92	0.95	0.93	0.93	0.92	0.90	0.90
DCAD^g^, mEq/kg	357	348	342	357	349	327	367	349	339	344
Total Ca: total P	0.60:1	1.29:1	2.00:1	2.71:1	3.40:1	0.40:1	0.85:1	1.32:1	1.79:1	2.25:1
Total Ca: STTD P	1.31:1	2.81:1	4.38:1	5.94:1	7.44:1	0.64:1	1.36:1	2.12:1	2.88:1	3.61:1
STTD Ca: STTD P	0.88:1	1.81:1	2.75:1	3.69:1	4.63:1	0.42:1	0.88:1	1.33:1	1.79:1	2.24:1

^a^All diets were formulated to have the following quantities of NE (kcal/kg), CP (%), AA (expressed as standardized ileal digestible AA; %), and minerals (%): NE, 2520; CP, 19.30; Arg, 1.20; His, 0.47; Ile, 0.72; Leu, 1.42; Lys, 1.23; Met, 0.40; Phe, 0.84; Thr, 0.73; Trp, 0.22; Val, 0.78; Na, 0.47; Cl, 0.33; and K, 0.90. Values for NE and standardized ileal digestibility, and concentration of macro minerals in ingredients were from NRC, 2012. Value for STTD Ca and STTD P are calculated value. (NRC [1], Stein et al. [2])

^b^The vitamin-micromineral premix provided the following quantities of vitamins and micro minerals per kilogram of complete diet: vitamin A as retinyl acetate, 11136 IU; vitamin D_3_ as cholecalciferol, 2208 IU; vitamin E as *DL*-alpha tocopheryl acetate, 66 IU; vitamin K as menadione dimethylprimidinol bisulfite, 1.42 mg; thiamin as thiamine mononitrate, 0.24 mg; riboflavin, 6.59 mg; pyridoxine as pyridoxine hydrochloride, 24 mg; vitamin B_12_, 0.03 mg; *D*-pantothenic acid as *D*-calcium pantothenate, 23.5 mg; niacin, 44.1 mg; folic acid, 1.59 mg; biotin, 0.44 mg; Cu, 20 mg as copper sulfate; Fe, 126 mg as iron sulfate; I, 1.26 mg as ethylenediamine dihydriodide; Mn, 60.2 mg as manganous sulfate; Se, 0.25 mg as sodium selenite and selenium yeast; and Zn, 124.9 mg as zinc sulfate

^c^AEE = acid hydrolyzed ether extract

^d^Phytate was calculated by dividing the phytate-bound P by 0.282 (Tran and Sauvant [16])

^e^Phytate-bound *P* values were calculated from analyzed phytate-bound P in the ingredients

^f^Non-phytate P was calculated as the difference between total P and phytate-bound P

^g^DCAD = dietary cation-anion difference. The DCAD was calculated as Na + K – Cl

**Table 3:** Ingredient composition and analyzed composition of experimental diets containing 0.42% and 0.50% standardized total tract digestible (STTD) P, as-fed basis^a^

Ingredient, %	0.42% STTD P					0.50% STTD P				
Total P, %:	0.63	0.63	0.63	0.63	0.63	0.71	0.71	0.71	0.71	0.71
Total Ca, %:	0.21	0.45	0.70	0.95	1.19	0.21	0.45	0.70	0.95	1.19
STTD Ca, %:	0.14	0.29	0.44	0.59	0.74	0.14	0.29	0.44	0.59	0.74
Corn	46.00	46.00	46.00	46.00	46.00	46.00	46.00	46.00	46.00	46.00
Soybean meal	32.50	32.50	32.50	32.50	32.50	32.50	32.50	32.50	32.50	32.50
Cornstarch	8.08	6.25	4.35	2.44	0.67	7.85	6.02	4.12	2.21	0.44
Lactose	10.00	10.00	10.00	10.00	10.00	10.00	10.00	10.00	10.00	10.00
Choice white grease	0.50	1.70	2.95	4.20	5.35	0.65	1.85	3.10	4.35	5.50
Calcium carbonate	0.16	0.79	1.44	2.10	2.72	0.16	0.79	1.44	2.10	2.72
Monocalcium phosphate	0.10	0.10	0.10	0.10	0.10	0.10	0.10	0.10	0.10	0.10
Monosodium phosphate	1.15	1.15	1.15	1.15	1.15	1.50	1.50	1.50	1.50	1.50
Sodium bicarbonate	0.27	0.27	0.27	0.27	0.27	–	–	–	–	–
*L*-Lys HCl	0.37	0.37	0.37	0.37	0.37	0.37	0.37	0.37	0.37	0.37
*DL*-Met	0.14	0.14	0.14	0.14	0.14	0.14	0.14	0.14	0.14	0.14
Thr	0.12	0.12	0.12	0.12	0.12	0.12	0.12	0.12	0.12	0.12
Val	0.01	0.01	0.01	0.01	0.01	0.01	0.01	0.01	0.01	0.01
Sodium chloride	0.40	0.40	0.40	0.40	0.40	0.40	0.40	0.40	0.40	0.40
Vitamin mineral premix^b^	0.20	0.20	0.20	0.20	0.20	0.20	0.20	0.20	0.20	0.20
Total	100.0	100.0	100.0	100.0	100.0	100.0	100.0	100.0	100.0	100.0
Analyzed composition										
GE, kcal/kg	3818	3869	3922	4014	4018	3845	3896	3928	4015	4008
DM, %	87.47	87.72	87.73	87.87	88.17	87.44	87.44	87.92	87.72	87.86
CP, %	19.33	19.01	17.91	18.78	18.43	19.59	18.86	18.89	18.41	18.81
Ash, %	4.82	5.44	5.54	6.71	7.82	5.80	5.51	6.45	7.16	8.06
AEE^c^, %	3.02	3.97	5.56	7.38	8.53	3.63	5.29	5.80	7.24	7.89
ADF, %	3.69	3.31	3.23	4.55	3.52	3.81	4.10	3.89	4.34	3.55
NDF, %	6.88	6.38	5.70	7.46	6.14	5.57	6.24	5.29	7.75	5.66
Ca, %	0.20	0.47	0.66	0.91	1.21	0.20	0.47	0.63	0.97	1.27
P, %	0.65	0.63	0.66	0.70	0.70	0.75	0.77	0.79	0.81	0.78
Phytate^d^, %	0.49	0.49	0.49	0.49	0.49	0.49	0.49	0.49	0.49	0.49
Phytate-bound P^e^, %	0.14	0.14	0.14	0.14	0.14	0.14	0.14	0.14	0.14	0.14
Non-phytate P^f^, %	0.51	0.49	0.52	0.56	0.56	0.61	0.63	0.65	0.67	0.64
Na, %	0.47	0.45	0.47	0.47	0.50	0.47	0.50	0.48	0.46	0.46
K, %	0.91	0.93	0.88	0.95	0.92	0.92	0.95	0.93	0.95	0.91
Cl, %	0.31	0.34	0.32	0.32	0.33	0.30	0.33	0.31	0.30	0.32
DCAD^g^, mEq/kg	349	339	341	360	358	355	369	359	358	347
Total Ca: total P	0.33:1	0.71:1	1.11:1	1.51:1	1.89:1	0.30:1	0.63:1	0.99:1	1.34:1	1.68:1
Total Ca: STTD P	0.50:1	1.07:1	1.67:1	2.26:1	2.83:1	0.42:1	0.90:1	1.40:1	1.90:1	2.38:1
STTD Ca: STTD P	0.33:1	0.69:1	1.05:1	1.40:1	1.76:1	0.28:1	0.58:1	0.88:1	1.18:1	1.48:1

^a^All diets were formulated to have the following quantities of NE (kcal/kg), CP (%), AA (expressed as standardized ileal digestible AA; %), and minerals (%): NE, 2520; CP, 19.30; Arg, 1.20; His, 0.47; Ile, 0.72; Leu, 1.42; Lys, 1.23; Met, 0.40; Phe, 0.84; Thr, 0.73; Trp, 0.22; Val, 0.78; Na, 0.47; Cl, 0.33; and K, 0.90. Values for NE and standardized ileal digestibility, and concentration of macro minerals in ingredients were from NRC, 2012.Values for STTD Ca and STTD P are calculated values (NRC [1], Stein et al. [2])

^b^The vitamin-micromineral premix provided the following quantities of vitamins and micro minerals per kilogram of complete diet: vitamin A as retinyl acetate, 11136 IU; vitamin D_3_ as cholecalciferol, 2208 IU; vitamin E as *DL*-alpha tocopheryl acetate, 66 IU; vitamin K as menadione dimethylprimidinol bisulfite, 1.42 mg; thiamin as thiamine mononitrate, 0.24 mg; riboflavin, 6.59 mg; pyridoxine as pyridoxine hydrochloride, 24 mg; vitamin B_12_, 0.03 mg; *D*-pantothenic acid as *D*-calcium pantothenate, 23.5 mg; niacin, 44.1 mg; folic acid, 1.59 mg; biotin, 0.44 mg; Cu, 20 mg as copper sulfate; Fe, 126 mg as iron sulfate; I, 1.26 mg as ethylenediamine dihydriodide; Mn, 60.2 mg as manganous sulfate; Se, 0.25 mg as sodium selenite and selenium yeast; and Zn, 124.9 mg as zinc sulfate

^c^AEE = acid hydrolyzed ether extract

^d^Phytate was calculated by dividing the phytate-bound P by 0.282 (Tran and Sauvant [16])

^e^Phytate-bound P values were calculated from analyzed phytate-bound P in the ingredients

^f^Non-phytate P was calculated as the difference between total P and phytate-bound P

^g^DCAD = dietary cation-anion difference. The DCAD was calculated as Na + K – Cl

### Growth performance, sample collection, and bone measurements

Pigs that weaned at 20 ± 2 days of age were allotted to experimental diets on d 20 post-weaning and allowed ad libitum access to feed for 21 d. The amount of feed offered was recorded daily and at the conclusion of the experiment, the amount of feed left in the feeders was recorded. Pig weights were recorded on d 1 and 21 when pigs had an average BW of 22.4 ± 3.3 kg.

On the last day of the experiment, 1 barrow in each pen with a BW closest to the average BW of the pen was euthanized via captive bolt stunning. Blood samples were collected from the vena cava using purple top vacutainers and immediately centrifuged and plasma samples were collected and stored at − 20 °C for analysis of Ca and P. The gastrointestinal tract was removed. Duodenal tissue was collected 30 cm distal to the pyloric spincter and ileal tissue samples were collected 30 cm anterior to the ileo-cecal valve. Ileal tissue was cut longitudinally, washed with PBS, and scraped with microscope slides to recover the mucosal layer. Duodenal tissue samples and the scraped mucosal layer from the ileum were snap-frozen in liquid N_2_ immediately after collection and stored at − 80 °C.

The right femur was collected and autoclaved at 125 °C for 55 min. Femurs were broken, dried, and soaked for 72 h in petroleum ether under a chemical hood to remove marrow and fat. Femurs were dried for 2 h at 135 °C and then ashed at 600 °C for 16 h.

### Sample analysis

Corn, soybean meal, lactose, calcium carbonate, monocalcium phosphate, monosodium phosphate, sodium bicarbonate, NaCl, and diets were analyzed for dry matter (DM) by oven drying at 135 °C for 2 h (Method 930.15, [11]) and ash (Method 942.05, [11]). Ingredients (except lactose) and diets were analyzed for Na and K by flame emission photometry (Method 956.01, [11]) and Cl by manual titration (Method 9.15.01, 943.01, [12]). Ingredients, diets, bone ash, and plasma samples were analyzed for Ca and P by inductively coupled plasma-optical emission spectrometry (Method 985.01 A, B, and D, [11]) after wet ash sample preparation [Method 975.03 B(b), [11]]. Corn, soybean meal, lactose, and diets were analyzed for gross energy (GE) using bomb calorimetry (Model 6400, Parr Instruments, Moline, IL). Corn, soybean meal, and diets were also analyzed for crude protein using the Kjeldahl method by quantifying N and using a conversion factor of 6.25 to calculate crude protein (CP; Method 984.13, [11]); a Kjeltec™ 8400 (FOSS, Eden Prairie, MN) was used. Acid hydrolyzed ether extract (AEE; Method 2003.06, [11]) was analyzed on an Ankom^XT15^ (Ankom Technology, Macedon, NY), and acid detergent fiber (ADF) and neutral detergent fiber (NDF) were analyzed using Ankom Technology method 12 and 13, respectively (Ankom 2000 Fiber Analyzer, Ankom Technology, Macedon, NY). Phytate-bound P was predicted in corn and soybean meal by near infra-red reflectance spectroscopy (ESC Standard Analytical Method, SAM120; AB Vista, Memphis, TN).

### RNA extraction and quantitative reverse-transcription PCR

The RNA was extracted from 30 ± 0.2 mg of frozen duodenal tissue and ileal mucosa using β-mercaptoethanol (Alfa Aesar, Tewksbury, MA) according to the RNeasy Mini Kit (QIAGEN, Germantown, MD) manufacturer’s instructions. Total RNA was quantified using a NanoDrop ND-1000 spectrophotometer (NanoDrop Technologies, Wilmington, DE). The RNA quality was determined using a Fragment Analyzer™ Automated CE System (Method DNF-471-33 - SS Total RNA 15 nt; Advanced Analytical, Ankeny, IA) and RNA samples with an RNA quality number greater than 7 were used for cDNA synthesis.

A portion of the RNA was diluted to 100 ng/μL with DNase/RNase-free water for cDNA synthesis as described by Vailati-Riboni et al. [13] using 4 and 2 μL of diluted RNA from duodenal tissue and ileal mucosa, respectively. The cDNA was then diluted 1:4 with DNase/RNase-free water, prior to qPCR analysis.

Quantitative PCR was performed using 4 μL of diluted cDNA combined with 6 μL of a mixture composed of 5 μL of SYBR Green master mix (PerfeCTa SYBR Green FastMix, ROX™; Quanta BioSciences, Beverly, MA), 0.4 μL each of 10 μmol/L forward and reverse primers, and 0.2 μL DNase/RNase free water in a MicroAmp™ Optical 384-Well Reaction Plate (Applied Biosystems, Foster City, CA). All samples were run in duplicate using a 7-point standard curve that was developed with samples run in triplicate. Reactions were performed in a QuantStudio™ 7 Flex Real-Time PCR System (Applied Biosystems, Foster City, CA) using the following conditions: 2 min at 50 °C, 10 min at 95 °C, 40 cycles of 15 s at 95 °C, and 1 min at 60 °C. The presence of a single PCR product was verified by the dissociation protocol. Data were analyzed using the QuantStudio™ Real-Time PCR Software (version 1.3; Applied Biosystems, Foster City, CA).

Three internal control genes, β-actin (*ACTB;* [14]), glyceraldehyde 3-phosphate dehydrogenase (*GAPDH*), and hydroxymethylbilane synthase (*HMBS*; [15]) were used to normalize the abundance of tested genes. Tested genes included S100 calcium binding protein G (*S100G*), transient receptor potential cation channel, subfamily V, member 6 (*TRPV6*), and ATPase, Ca^2+^ transporting, plasma membrane-1 (*ATP2B1*) for the duodenal tissue; and occludin (*OCLN*), zonula occludens-1 (*ZO1*), and claudin-1 (*CLDN1*) for both the duodenal tissue and ileal mucosa. The *S100G*, *TRPV6*, and *ATP2B1* genes are important for transcellular absorption of Ca, whereas *OCLN* and *CLDN1* are tight junction proteins that are important for paracellular transport of Ca. Primers (Additional file 1: Table S1) were commercially synthesized by Integrated DNA Technologies (Skokie, IL).

### Calculations and statistical analyses

The percentages of phytate in corn, soybean meal, and diets were calculated by dividing the analyzed phytate-bound P by 0.282 [16], and non-phytate P was calculated by subtracting the amount of phytate-bound P from total P. Dietary cation-anion difference (DCAD) was calculated using the following equation [3]:

DCAD, mEq/kg = [(Na × 10000)/23] + [(K × 10000)/39] – [(Cl × 10000)/35.5],

where Na, K, and Cl were expressed as percentages of the diet. The average daily gain (ADG), average daily feed intake (ADFI), and gain to feed (G:F) were calculated for pigs fed each diet and concentrations of bone Ca and bone P in grams per femur were calculated by multiplying the total quantity of bone ash by the percentage of Ca and P in the bone ash. The final data from the abundance of tested genes were normalized using the geometric mean of the 3 internal control genes and the real-time quantitative PCR data were log_2_ transformed before statistical analysis to obtain a normal distribution.

Normality of residuals and assumptions of the model were tested using PROC GPLOT and influence options of SAS (SAS Inst. Inc., Cary, NC). Data for growth performance, concentration and percentage of bone ash, bone Ca, and bone P, concentration of Ca and P in plasma, and abundance of genes were analyzed using the PROC MIXED of SAS with the experimental unit being the pen. The fixed effects of the model were dietary concentration of STTD Ca, dietary concentration of STTD P, and the interaction between STTD Ca and STTD P; the random effect was block. Effects of dietary STTD Ca, STTD P, and the interaction between STTD Ca and STTD P were considered significant at *P* ≤ 0.10. The LSMEANS procedure was used to calculate mean values for treatments. If the interaction or the main effects were significant, the software NLREG version 6.5 [17] was used to determine parameter estimates for the second-order response surface model to increasing concentrations of STTD Ca and STTD P as described by Khuri and Cornell [18]. Parameter estimates of the model that were not significant (*P* > 0.10) and were not included in a significant interaction were removed from the model and the estimates were recalculated. The surface response full model was:

*Y* = *a* + *b* × STTD Ca + *c* × STTD Ca^2^ + *d* × STTD P + *e* × STTD P^2^ + *f *× STTD Ca × STTD P + *g* × STTD Ca^2^ × STTD P + *h* × STTD Ca × STTD P^2^ + *i *× STTD Ca^2^ × STTD P^2^,

where *Y* is the dependent variable, *a* is the intercept, *b*, *c*, *d*, *e*, *f*, *g*, *h*, and *i* are the coefficients, and STTD Ca and STTD P are the percentage concentrations of dietary STTD Ca and STTD P. By solving the above equation, the percentage concentrations of STTD Ca at the maximum response values were calculated using the following equation:

STTD Ca _max_ (%) = [−(*h* × STTD P^2^ + *f* × STTD P + *b*)] / [2 × (*i* × STTD P^2^ + *g* × STTD P + *c*)],

where STTD Ca _max_ is the percentage concentration of STTD Ca at the maximum response and STTD P is the percentage concentration of STTD P in the diet. The maximum response values were, therefore, calculated for the variables of interest using the respective model equations with the concentrations of STTD Ca at the maximum response for each concentration of STTD P.

## Results

**Fig. 1:**
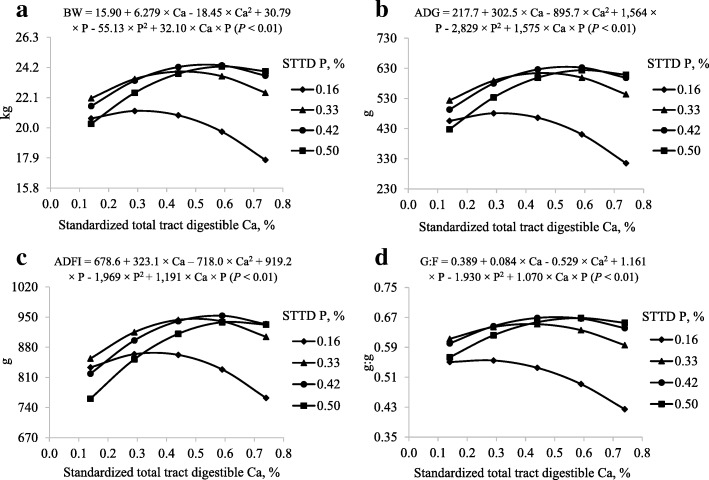
Models and predicted values, based on the interaction between Ca and P (*P* < 0.01), for (**a**) final body weight (BW), (**b**) average daily gain (ADG), (**c**) average daily feed intake (ADFI), and (**d**) gain to feed ratio (G:F) at d 21 in pigs fed diets containing from 0.14% to 0.74% standardized total tract digestible (STTD) Ca and from 0.16% to 0.50% STTD of P

Overall, there was 1.9% mortality in the experiment and the removed pigs were from 6 different diets and values for ADG, ADFI, and G:F (Table 4) of these pens were adjusted as previously explained [19]. The remaining animals consumed their diets without apparent problems and remained healthy throughout the experiment. The model to predict final BW, ADG, ADFI, and G:F was reduced because only the interaction (*P* < 0.01) between STTD Ca and STTD P was significant. The predicted maximum BW at STTD P concentrations of 0.16%, 0.33%, 0.42%, and 0.50% were 21.18, 23.92, 24.40, and 24.27 kg at STTD Ca concentrations of 0.31%, 0.46%, 0.54%, and 0.61% respectively. These values correspond to STTD Ca:STTD P ratios of 1.93:1, 1.39:1, 1.28:1, and 1.21:1 (Fig. 1a). The predicted maximum ADG at STTD P concentrations of 0.16%, 0.33%, 0.42%, and 0.50% were 481, 614, 635, and 624 g at STTD Ca concentrations of 0.31%, 0.46%, 0.54%, and 0.61% respectively, corresponding to STTD Ca:STTD P ratios of 1.93:1, 1.39:1, 1.28:1, and 1.22:1 (Fig. 1b). The predicted maximum ADFI at STTD P concentrations of 0.16%, 0.33%, 0.42%, and 0.50% were 867, 946, 953, and 940 g at STTD Ca concentrations of 0.36%, 0.50%, 0.57%, and 0.64% respectively, which correspond to STTD Ca:STTD P ratios of 2.24:1, 1.51:1, 1.36:1, and 1.28:1 (Fig. 1c). The predicted maximum G:F at STTD P concentrations of 0.16%, 0.33%, 0.42%, and 0.50% were 0.556, 0.652, 0.671, and 0.668 g:g at STTD Ca concentrations of 0.24%, 0.41%, 0.50%, and 0.59% respectively. These values correspond to STTD Ca:STTD P ratios of 1.51:1, 1.25:1, 1.20:1, and 1.17:1 (Fig. 1d).

**Table 4:** Least squares means for growth performance of pigs fed diets containing different concentrations of standardized total tract digestible (STTD) Ca and STTD P for 21 d^a^

Item	STTD Ca, %				
	0.14	0.29	0.44	0.59	0.74
Initial BW, kg^b^					
0.16% STTD P	11.13	10.98	11.21	11.06	11.02
0.33% STTD P	10.89	10.98	11.01	11.17	11.09
0.42% STTD P	11.11	11.12	11.12	11.14	10.95
0.50% STTD P	11.15	11.09	11.05	11.19	11.08
Final BW, kg^c^					
0.16% STTD P	20.87	20.91	20.77	19.36	18.11
0.33% STTD P	22.07	23.78	23.79	23.47	22.61
0.42% STTD P	20.69	24.50	23.67	24.00	23.47
0.50% STTD P	20.04	22.80	23.71	24.27	24.01
ADG, g^d^					
0.16% STTD P	465	473	455	395	337
0.33% STTD P	530	609	621	586	548
0.42% STTD P	451	637	598	612	596
0.50% STTD P	413	557	599	622	615
ADFI, g^e^					
0.16% STTD P	848	849	864	808	791
0.33% STTD P	871	948	931	933	914
0.42% STTD P	763	978	895	958	928
0.50% STTD P	752	869	925	940	936
G:F, g:g^f^					
0.16% STTD P	0.549	0.556	0.527	0.488	0.425
0.33% STTD P	0.610	0.645	0.656	0.628	0.601
0.42% STTD P	0.592	0.654	0.671	0.640	0.643
0.50% STTD P	0.549	0.643	0.649	0.663	0.658

^a^Data are least squares means of 8 observations with the exception of diet 0.44% STTD Ca and 0.33% STTD P for ADG (*n* = 7)

^b^Standard error of the within treatment least squares means = 0.51

^c^Standard error of the within treatment least squares means = 0.80

^d^Standard error of the within treatment least squares means = 0.02

^e^Standard error of the within treatment least squares means = 0.04

^f^Standard error of the within treatment least squares means = 0.01

**Fig. 2:**
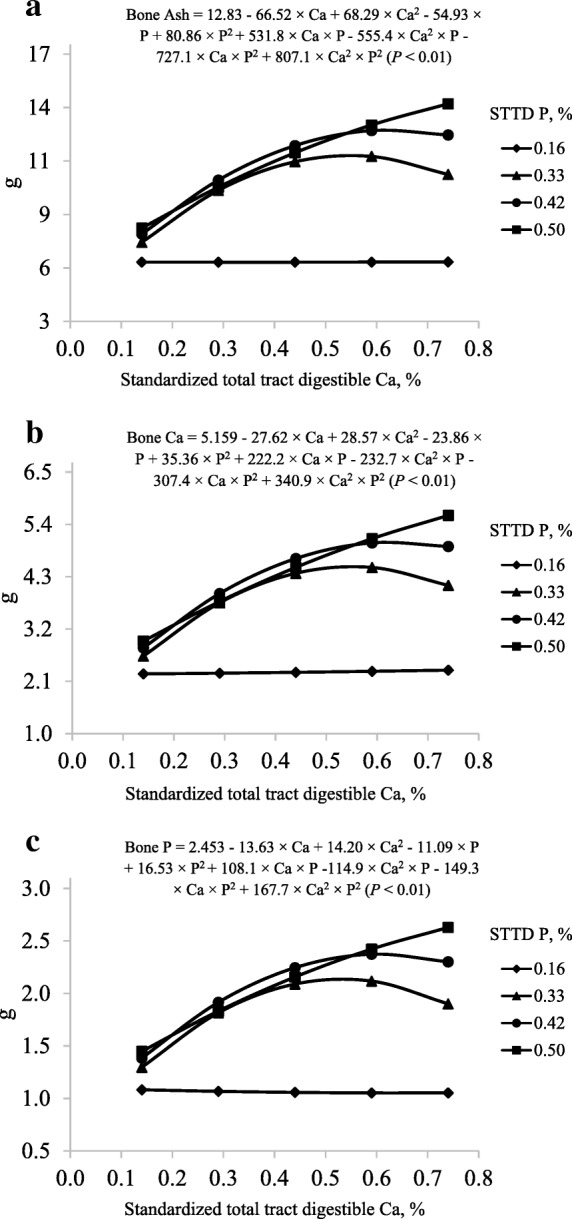
Models and predicted values, based on the interactions between Ca and P (*P* < 0.01), for (**a**) bone ash, (**b**) bone Ca, and (**c**) bone P in grams per femur (g) at d 21 in pigs fed diets containing from 0.14% to 0.74% standardized total tract digestible (STTD) Ca and from 0.16% to 0.50% STTD of P

The full model was used to predict the concentration of bone ash, bone Ca, and bone P in g per femur (interactions, *P* < 0.01; Table 5). The predicted maximum bone ash at STTD P concentrations of 0.33%, 0.42%, and 0.50% were 11.68, 13.03, and 15.68 g at STTD Ca concentrations of 0.55%, 0.63%, and 1.15%, respectively. These values correspond to STTD Ca:STTD P ratios of 1.66:1, 1.50:1, and 2.30:1 (Fig. 2a). The predicted maximum bone Ca at STTD P concentrations of 0.33%, 0.42%, and 0.50% were 4.51, 5.03, and 6.41 g at STTD Ca concentrations of 0.55%, 0.64%, and 1.31%, respectively, corresponding to STTD Ca:STTD P ratios of 1.67:1, 1.52:1, and 2.62:1 (Fig. 2b). The predicted maximum bone P at STTD P concentrations of 0.33%, 0.42%, and 0.50% were 2.14, 2.38, and 2.89 g at STTD Ca concentrations of 0.53%, 0.61%, and 1.19%, respectively, which correspond to STTD Ca:STTD P ratios of 1.61:1, 1.45:1, and 2.38:1 (Fig. 2c). However, maximum bone ash, bone Ca, and bone P in grams per femur at STTD P concentration of 0.16% were not estimated because of a lack of response to increasing concentrations of dietary STTD Ca.

**Table 5:** Least squares means for concentration (grams per femur) and percentage of bone ash, bone Ca, and bone P in pigs fed diets containing different concentrations of standardized total tract digestible (STTD) Ca and STTD P for 21 d^a^

Item	STTD Ca, %				
	0.14	0.29	0.44	0.59	0.74
Bone ash, g per femur^b^					
0.16% STTD P	5.97	5.85	6.52	5.63	6.24
0.33% STTD P	7.08	10.63	11.20	11.12	10.76
0.42% STTD P	7.29	10.49	11.42	13.97	12.75
0.50% STTD P	8.13	9.89	11.84	13.44	14.19
Bone Ca, g per femur^c^					
0.16% STTD P	2.20	2.17	2.46	2.12	2.39
0.33% STTD P	2.61	4.01	4.35	4.27	4.12
0.42% STTD P	2.73	3.96	4.38	5.46	4.95
0.50% STTD P	3.03	3.76	4.46	5.18	5.50
Bone P, g per femur^d^					
0.16% STTD P	1.06	1.03	1.13	0.98	1.08
0.33% STTD P	1.29	1.95	2.08	2.00	1.92
0.42% STTD P	1.35	1.92	2.13	2.59	2.30
0.50% STTD P	1.49	1.83	2.15	2.45	2.60
Bone ash, %^e^					
0.16% STTD P	39.85	40.38	42.05	40.12	43.68
0.33% STTD P	43.37	48.08	48.95	49.25	48.63
0.42% STTD P	42.93	48.49	49.95	51.92	51.47
0.50% STTD P	45.12	47.95	49.92	52.58	53.12
Bone Ca, %^f^					
0.16% STTD P	37.11	36.98	37.67	37.82	38.12
0.33% STTD P	36.79	37.73	38.80	38.43	38.32
0.42% STTD P	37.41	37.68	38.33	38.92	38.87
0.50% STTD P	37.20	38.10	37.61	38.51	38.62
Bone P, %^g^					
0.16% STTD P	17.78	17.64	17.38	17.58	17.27
0.33% STTD P	18.25	18.33	18.62	17.96	17.86
0.42% STTD P	18.49	18.25	18.63	18.49	18.08
0.50% STTD P	18.34	18.55	18.17	18.22	18.29
Ca:P in bone^h^					
0.16% STTD P	2.09	2.09	2.17	2.15	2.21
0.33% STTD P	2.01	2.06	2.08	2.14	2.15
0.42% STTD P	2.02	2.06	2.06	2.10	2.15
0.50% STTD P	2.03	2.05	2.07	2.11	2.11

^a^Data are least squares means of 8 observations with the exception of diet 0.14% STTD Ca and 0.16% STTD P (*n* = 7)

^b^Standard error of the within treatment least squares means = 0.54

^c^Standard error of the within treatment least squares means = 0.23

^d^Standard error of the within treatment least squares means = 0.10

^e^Standard error of the within treatment least squares means = 0.82

^f^Standard error of the within treatment least squares means = 0.57

^g^Standard error of the within treatment least squares means = 0.26

^h^Standard error of the within treatment least squares means = 0.02

**Fig. 3:**
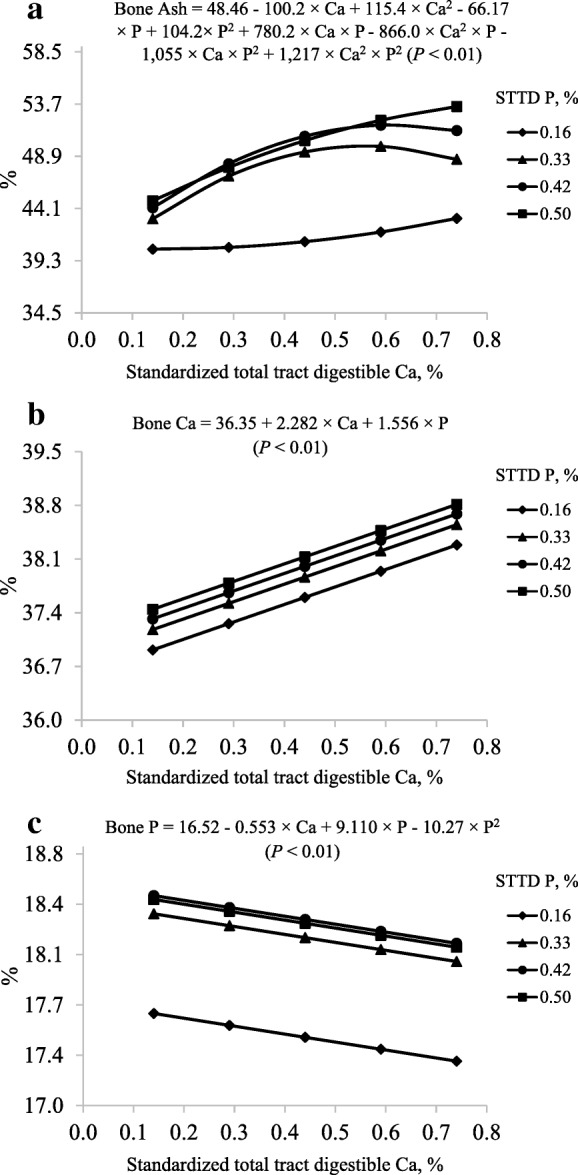
Models and predicted values, based on the interactions between Ca and P (*P* < 0.01), for percentage of (**a**) bone ash (%), based on the linear effect of Ca (*P* < 0.01) and P (*P* < 0.10), for percentage of (**b**) bone Ca, and based on the linear effect of Ca and P (*P* < 0.05) and the quadratic effect of P (*P* < 0.05), for percentage of (**c**) bone P at d 21 in pigs fed diets containing from 0.14% to 0.74% standardized total tract digestible (STTD) Ca and from 0.16% to 0.50% STTD of P

The model to predict percentage of bone ash was not reduced (interactions, *P* < 0.01) and the predicted maximum bone ash at STTD P concentrations of 0.33%, 0.42%, and 0.50% were 49.84%, 51.79%, and 54.27% at STTD Ca concentrations of 0.56%, 0.62%, and 0.98%, respectively. These values correspond to STTD Ca:STTD P ratios of 1.70:1, 1.47:1, and 1.97:1 (Fig. 3a). However, a maximum percentage of bone ash at STTD P concentration of 0.16% was not estimated because of a lack of a positive quadratic response to increasing dietary STTD Ca. The reduced model to predict percentage of bone Ca only contained the linear (*P* < 0.05) STTD Ca and STTD P terms (Fig. 3b); whereas, the reduced model to predict percentage of bone P contained the linear (*P* < 0.05) STTD Ca and STTD P terms and the quadratic (*P* < 0.05) STTD P term (Fig. 3c).

**Fig. 4:**
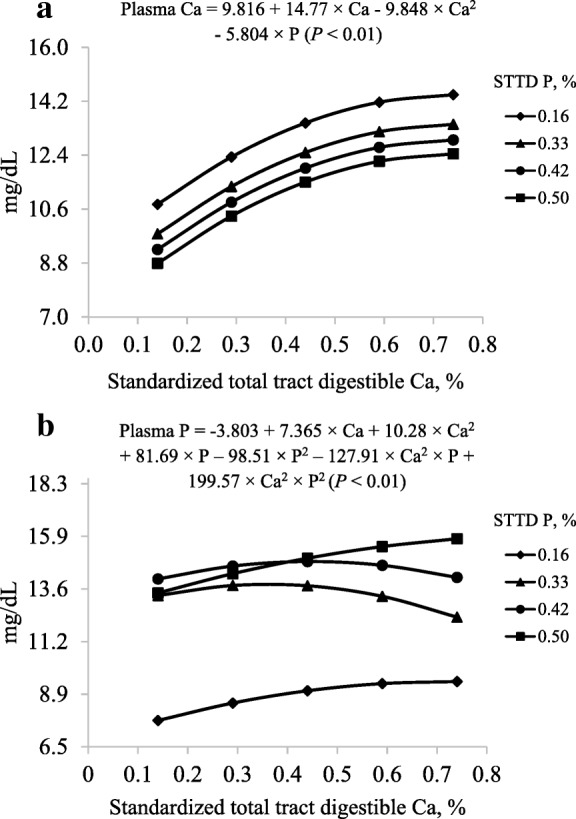
Models and predicted values, based on the linear and quadratic effect of Ca (*P* < 0.01) and the linear effect of P (*P* < 0.01), for (**a**) plasma Ca (mg/dL) and based on the interactions between Ca and P (*P* < 0.01), (**b**) for plasma P (mg/dL) at d 21 in pigs fed diets containing from 0.14% to 0.74% standardized total tract digestible (STTD) Ca and from 0.16% to 0.50% STTD of P

In the model to predict the concentration of plasma Ca in milligrams per deciliter, there were no interactions between STTD Ca and STTD P (Table 6). The reduced model only contained the linear and quadratic (*P* < 0.01) STTD Ca terms and the linear (*P* < 0.01) STTD P term (Fig. 4a).

**Table 6:** Least squares means for concentration of Ca and P in plasma of pigs fed diets containing different concentrations of standardized total tract digestible (STTD) Ca and STTD P for 21 d^a^

Item	STTD Ca, %				
	0.14	0.29	0.44	0.59	0.74
Plasma Ca, mg/dL^b^					
0.16% STTD P	11.13	12.07	13.78	13.65	15.42
0.33% STTD P	9.37	11.68	12.35	12.96	13.47
0.42% STTD P	8.07	11.33	11.67	12.50	13.20
0.50% STTD P	9.40	11.03	12.07	11.78	12.14
Plasma P, mg/dL^c^					
0.16% STTD P	8.17	8.36	8.85	9.08	9.75
0.33% STTD P	12.78	14.67	14.26	12.18	12.48
0.42% STTD P	13.73	14.16	15.28	15.21	14.11
0.50% STTD P	13.51	14.66	15.05	14.59	16.29

^a^Data are least squares means of 7 or 8 observations

^b^Standard error of the within treatment least squares means = 0.42

^c^Standard error of the within treatment least squares means = 0.59

For concentration of plasma P in milligrams per deciliter, the model was reduced because the interactions between linear STTD Ca and STTD P terms and between linear STTD Ca and quadratic STTD P terms were not significant; however, interactions (*P* < 0.01) between quadratic STTD Ca and linear STTD P terms and between quadratic STTD Ca and STTD P terms were observed (Fig. 4b).

**Fig. 5:**
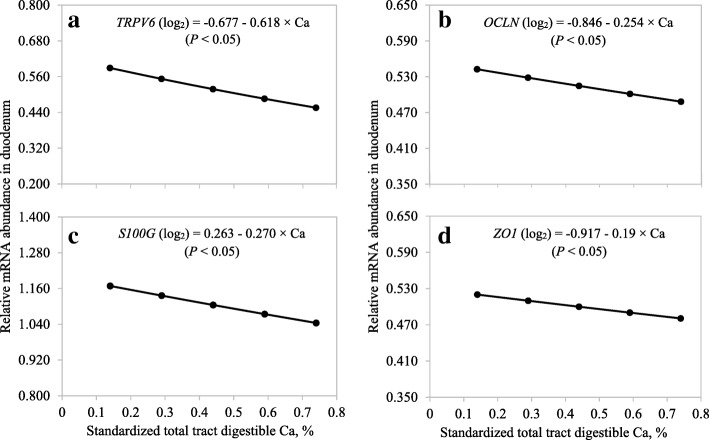
Predicted values, based on the linear effect of Ca (*P* < 0.05), for mRNA abundance of (**a**) transient receptor potential cation channel, subfamily V, member 6 (*TRPV6*), (**b**) occludin (*OCLN*), (**c**) S100 calcium binding protein G (*S100G*), and (**d**) zonula occludens-1 (*ZO1*) in the duodenum of pigs fed diets containing from 0.14% to 0.74% standardized total tract digestible (STTD) Ca

**Fig. 6:**
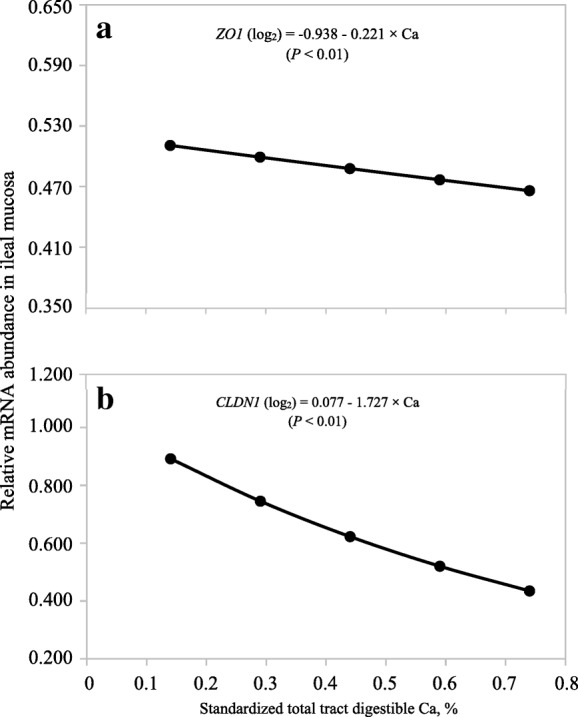
Predicted values, based on the linear effect of Ca (*P* < 0.01), for mRNA abundance of (**a**) zonula occludens-1 (*ZO1*) and (**b**) claudin-1 (*CLDN1*) in the ileum of pigs fed diets containing from 0.14% to 0.74% standardized total tract digestible (STTD) Ca

Neither Ca nor P could be used to predict the abundance of *ATP2B1* or *CLDN1* in the duodenum (Table 7) or the abundance of *OCLN* in the ileum (Table 8). The model to predict the abundance of *TRPV6*, *S100G*, *OCLN*, and *ZO1* in the duodenum and the abundance of *CLDN* and *ZO1* in the ileum only contained the linear (*P* < 0.05) STTD Ca term (Fig. 5 and Fig. 6).

**Table 7:** Least squares means (log_2_-backtransformed) for mRNA abundance in the duodenum of pigs fed diets containing different concentrations of standardized total tract digestible (STTD) Ca and STTD P for 21 d^a^

Item	STTD Ca, %				
	0.14	0.29	0.44	0.59	0.74
*TRPV6* ^b^					
0.16% STTD P	0.461	0.478	0.438	0.447	0.425
0.33% STTD P	0.526	0.632	0.587	0.479	0.466
0.42% STTD P	0.717	0.522	0.539	0.474	0.585
0.50% STTD P	0.749	0.633	0.601	0.519	0.431
*S100G* ^c^					
0.16% STTD P	1.175	1.126	0.968	1.050	1.041
0.33% STTD P	1.182	1.175	1.208	0.983	1.091
0.42% STTD P	1.117	1.116	1.106	0.955	1.109
0.50% STTD P	1.207	1.017	1.112	1.053	1.162
*ATP2B1* ^d,e^					
0.16% STTD P	0.426	0.394	0.388	0.409	0.390
0.33% STTD P	0.381	0.410	0.435	0.399	0.467
0.42% STTD P	0.422	0.389	0.376	0.412	0.419
0.50% STTD P	0.435	0.389	0.374	0.422	0.468
*OCLN* ^f^					
0.16% STTD P	0.550	0.518	0.470	0.490	0.475
0.33% STTD P	0.526	0.523	0.521	0.483	0.510
0.42% STTD P	0.548	0.511	0.548	0.471	0.505
0.50% STTD P	0.580	0.512	0.502	0.488	0.515
*ZO1* ^g^					
0.16% STTD P	0.534	0.542	0.504	0.510	0.486
0.33% STTD P	0.535	0.521	0.501	0.500	0.458
0.42% STTD P	0.511	0.508	0.537	0.493	0.491
0.50% STTD P	0.525	0.497	0.448	0.468	0.519
*CLDN1* ^h,i^					
0.16% STTD P	0.656	0.338	0.338	0.323	0.588
0.33% STTD P	0.338	0.495	0.290	0.287	0.160
0.42% STTD P	0.460	0.304	0.470	0.385	0.298
0.50% STTD P	0.349	0.522	0.313	0.314	0.359

^a^Data are least squares means of 6, 7, or 8 observations

^b^Standard error of the within treatment least squares means = 0.10

^c^Standard error of the within treatment least squares means = 0.09

^d^Results indicated that abundance of *ATP2B1* could not be predicted using STTD Ca or STTD P

^e^Standard error of the within treatment least squares means = 0.04

^f^Standard error of the within treatment least squares means = 0.04

^g^Standard error of the within treatment least squares means = 0.03

^h^Results indicated that abundance of *CLDN1* could not be predicted using STTD Ca or STTD P

^i^Standard error of the within treatment least squares means = 0.10

**Table 8:** Least squares means (log_2_-backtransformed) for mRNA abundance in the ileum of pigs fed diets containing different concentrations of standardized total tract digestible (STTD) Ca and STTD P for 21 d^a^

Item	STTD Ca, %				
	0.14	0.29	0.44	0.59	0.74
*OCLN* ^b,c^					
0.16% STTD P	0.570	0.574	0.507	0.514	0.535
0.33% STTD P	0.665	0.455	0.678	0.529	0.598
0.42% STTD P	0.474	0.502	0.597	0.553	0.522
0.50% STTD P	0.578	0.541	0.432	0.596	0.557
*ZO1* ^d^					
0.16% STTD P	0.522	0.544	0.464	0.503	0.479
0.33% STTD P	0.518	0.502	0.499	0.472	0.453
0.42% STTD P	0.521	0.458	0.525	0.473	0.452
0.50% STTD P	0.476	0.478	0.481	0.504	0.440
*CLDN1* ^e^					
0.16% STTD P	1.105	0.476	0.700	0.578	0.432
0.33% STTD P	0.526	0.850	0.361	0.425	0.488
0.42% STTD P	1.275	0.430	0.667	0.523	0.444
0.50% STTD P	1.493	1.114	0.821	0.560	0.620

^a^Data are least squares means of 6, 7, or 8 observations

^b^Results indicated that abundance of *OCLN* could not be predicted using STTD Ca or STTD P

^c^Standard error of the within treatment least squares means = 0.26

^d^Standard error of the within treatment least squares means = 0.08

^e^Standard error of the within treatment least squares means = 0.58

## Discussion

The analyzed concentrations of Ca, P, Na, Cl, and K in corn, soybean meal, calcium carbonate, monocalcium phosphate, monosodium phosphate, sodium bicarbonate, and NaCl was within the range of previously published values [20–23]. Diets were formulated using NRC [1] values for Ca and P in ingredients. This may explain the differences between calculated and analyzed values in some diets. However, the objective of obtaining differences in both Ca and P among diets was met. The calculated values for DCAD, which ranged from 327 to 369 mEq/kg, were within the range of DCAD values that support maximum growth performance in young pigs (200 to 500 mEq/kg; [24]).

The diet containing 0.33% STTD P and 0.70% total Ca was formulated to meet NRC requirements for Ca and P. However, it is acknowledged that NRC requirements are requirement estimates and that these estimates are related to a presumed NE intake and a presumed growth rate of pigs. Pigs fed some of the diets in this experiment had reduced growth performance relative to NRC estimates, which was expected due to the low provisions of Ca and P in these diets. It has been suggested that the NRC estimated requirement for STTD P is less than the requirement of modern pigs [25]. However, it is possible that this conclusion was reached because dietary Ca was above the estimated requirement, which is expected to result in increased estimates for STTD P [3–5].

In an experiment that aimed at determining STTD Ca requirements by 11- to 25-kg pigs, a fixed concentration of 0.36% STTD P and 6 increasing concentrations of STTD Ca, from 0.32% to 0.72% were used [6]. Results indicated that increasing dietary STTD Ca from 0.32% to approximately 0.56% did not affect ADG and G:F, but providing dieatry STTD Ca above approximately 0.56% reduced ADG and G:F. This response pattern prevented the estimation of a STTD Ca concentration that maximized growth performance. Therefore, in this study, different concentrations of STTD P (below, at, and above the requirement; [1]) were used to estimate STTD Ca requirements at different concentrations of STTD P. The reason for including 4 concentrations of STTD P was that in a previous experiment [4], where only 3 STTD P concentrations were used, the response to increasing dietary Ca on growth performance was different if STTD P was at or below the requirement than if STTD P was above the requirement. Therefore, in the current experiment, 2 concentrations of STTD P that were above the requirement were used.

The surface response model was chosen following recommendations about the use of this model in factorial experiments with independent variables such as quantities of dietary components [26, 27]. By using this model, interactions between the independent and response variables can be quantified to estimate the values for the independent variables that maximize the response [18].

Results obtained in the current experiment indicated a detrimental effect of increased dietary Ca on growth performance if diets were deficient or marginal in P. This observation concurs with data previously reported [6]. However, the negative effect of Ca was ameliorated by including P above the requirement, and this was also observed in research conducted in poultry [28, 29] and in pigs [3–5, 30]. The reason for the negative response in growth performance by increasing concentrations of dietary Ca is likely the formation of Ca-P complexes in the intestinal tract due to excess Ca, which causes a reduction in P digestibility [31–34]. As a consequence, increased dietary Ca induced a P-deficiency even if dietary P was at the requirement. The fact that inclusion of P above the requirement reduces the negative effect of increasing concentrations of dietary Ca on growth performance, further indicates that the detrimental effect of excess Ca may be a result of P being insufficiently absorbed. The negative effect of excess Ca on the digestibility of P has been previously observed in pigs [32] and broiler chickens [35].

The observation that the concentration of STTD Ca that maximized BW, ADG, and G:F ranged from 0.24% to 0.31% corresponding to STTD Ca:STTD P ratios from 1.51:1 to 1.93:1 if dietary P was below the requirement, indicates that if STTD P is deficient in the diet, the concentration of STTD Ca also needs to be supplied below the requirement to avoid a reduction in growth performance. In contrast, if STTD P was above the requirement, dietary Ca became the limiting factor for growth performance, as demonstrated by the gradual improvement in growth performance as dietary Ca increased from 30% to 140% of the requirement. The observation that if STTD P concentrations were above the requirement, a concentration of STTD Ca to maximize BW, ADG, and G:F that ranged from 0.50% to 0.61%, with corresponding STTD Ca:STTD P ratios from 1.17:1 to 1.28:1 was needed, indicates that if the concentration of STTD P is above the requirement, STTD Ca also needs to be supplied above the requirement as also observed in heavier pigs [4]. This observation is relevant in finishing pigs fed diets with high concentration of P-rich coproducts such as distiller’s dried grains with solubles, because such diets often contain P in concentrations that exceed the requirement. The current data indicate that in this situation, dietary Ca also needs to be increased to maximize growth performance.

The reason the optimum dietary STTD Ca:STTD P ratio is reduced as dietary P is increased from below to above the requirement likely is a result of the fact that if P is supplied below the requirement, tissue accumulation of P in mussle is reduced and the ratio of dietary P used for soft tissue synthesis is less than if P is supplied at or above the requirement. As a consequence, if P is at the requirement, a greater proportion of dietary P is used for soft tissue synthesis and less for bone tissue synthesis and the required quantity of Ca to support bone tissue synthesis is less per unit of P supplied.

The fact that if STTD P was included at the requirement, BW, ADG, and G:F was maximized at 0.41% to 0.46% STTD Ca, indicates that the current NRC [1] requirement for total Ca (0.70%) likely is accurate because this level of total Ca corresponds to 0.44% STTD Ca. However, this level of dietary Ca will maximize growth performance only if dietary STTD P is at the requirement (i.e., 0.33%). The corresponding STTD Ca:STTD P ratios that maximize growth performance ranged from 1.25:1 to 1.39:1, which supports our hypothesis that a STTD Ca:STTD P ratio less than 1.40:1 maximizes growth performance for 11- to 25-kg pigs. These data also support the previous data indicating that a STTD Ca:STTD P ratio greater than 1.50:1 and 1.39:1 is detrimental to ADG and G:F, respectively [6].

The observation that at low dietary STTD Ca, feeding STTD P above the requirement resulted in reduced ADG and G:F compared with pigs fed STTD P at the requirement indicates that dietary P may chelate some of the dietary Ca in the intestinal tract and prevent absorption. As a consequence, in situations where Ca is the limiting nutrient, as is the case if Ca deficient diets are fed, excess dietary P appears to be detrimental for ADG and G:F.

The femur was used to estimate bone mineralization because it is believed to be an accurate indicator of the body mineral content of pigs [36]. The observation that bone ash, bone Ca, and bone P in grams per femur were maximized at STTD Ca:STTD P ratios between 1.61:1 to 1.67:1 if STTD P was at the requirement is in agreement with data demonstrating that a greater STTD Ca:STTD P ratio is required to maximize bone ash than to maximize growth performance [3–5]. The implication of this observation is that if the Ca requirement for growth has been met, Ca along with P are used to synthesize more skeletal tissue. However, if the dieatry STTD Ca:STTD P ratio is 1.35:1, the concentration of bone ash would be 97% of that observed if a ratio of 1.66:1 is used. This observation indicates that formulating diets based on STTD Ca:STTD P ratios that maximize growth performance does not dramatically affect bone mineralization.

Because the concentration (grams per femur) of bone Ca and bone P was calculated by multiplying the concentration of bone ash by the percentage of Ca and P in bone ash, the responses for quantities of bone Ca and bone P in grams per femur were similar to results observed for bone ash. The fact that bone ash (grams per femur) was not affected by dietary Ca if STTD P was below the requirement, indicates that P deficiency was limiting bone deposition and addition of extra Ca did not ameliorate this situation. In contrast, if the concentration of STTD P was at or above the requirement [1], Ca deficiency limited bone deposition; thus, increasing STTD Ca increased bone ash (grams per femur) up to the point were P became the limiting factor. This point was reached at approximately 0.50% and 0.60% STTD Ca if STTD P was at the requirement or at 130% of the requirement, respectively. For STTD P at 150% of the requirement, it appeared that STTD Ca up to 0.74% was the limiting nutrient for maximum bone tissue synthesis. These responses in the quantity of bone ash (grams per femur) at changing dietary Ca and P concentrations demonstrate the interaction between these 2 minerals in the skeletal tissue and the need for both Ca and P for bone tissue synthesis, which has also been previously demonstrated [37].

The increased percentage of bone ash in pigs fed diets deficient in P as more Ca was included in the diet indicates that the lack of response in bone ash (grams per femur) to increasing concentrations of Ca was possibly a result of the size of the bones, which were smaller in pigs fed high Ca diets compared with pigs fed low Ca diets. By contrast, in pigs fed diets with adequate or excess P, the response in percentage of bone ash was similar to that observed for concentration of bone ash (grams per femur). Calcium was limiting bone mineralization in low Ca diets, but P became limiting in high Ca diets, except at the highest concentration of P.

The Ca to P ratio in bone ash ranged from 2.01:1 to 2.21:1; these values are in agreement with reported data [3–5] and are close to the ratio of 2.1:1 that is needed to form hydroxyapatite crystals [Ca_10_(PO_4_)_6_(OH)_2_] [37, 38]. Although the concentration of Ca and P in bone ash is relatively constant, data from this study indicate that percentages of Ca and P in bone ash are responsive to the level of Ca in the diet. Regardless of the level of dietary P, the percentage of Ca in bone ash was increased by increasing concentrations of dietary Ca; whereas, increased dietary Ca decreased the percentage of P in bone ash, indicating a negative effect of Ca on P deposition. However, the changes in the percentage of Ca and P in bone ash, although significant, were relatively small.

As mentioned, if STTD P is at the requirement, the STTD Ca:STTD P ratio that maximizes growth performance declines as pig BW increases. However, it was also observed that the STTD Ca:STTD P ratio needed to maximize bone ash (grams per femur) increases as the animal becomes heavier. Bone ash was maximized at STTD Ca:STTD P ratios of 1.66:1, 1.81:1, 2.03:1, and 2.33:1 in pigs from 11 to 25 kg, 25 to 50 kg [3], 50 to 85 kg [4], and 100 to 130 kg [5], respectively, if STTD P was provided at the requirement. A possible explanation for this observation is that young pigs were supplied sufficient Ca through the milk while they were nursing and as a consequence when this experiment started, bone ash was already maximized and a dietary Ca:P ratio below that in bones was sufficient to maintain bone composition. In contrast, growing and finishing pigs are supplied diets that are deficient in Ca (in relation to what is needed to maximize bone ash), which limits bone tissue synthesis. Therefore, older pigs have greater capacity to increase skeletal tissue synthesis than younger pigs and a greater dietary Ca:P ratio is, therefore, needed to maximize bone ash. It is also possible that because P is needed for lean tissue deposition, which is decreasing as pigs get older, less P is needed relative to Ca, which may also contribute to an increase in the STTD Ca: STTD P ratio. Further research is needed to confirm this hypothesis.

Results from plasma analysis indicated that plasma Ca concentration is responsive to dietary Ca. The observation that as STTD P increased, plasma Ca was reduced indicates that if P is available, more bone tissue synthesis can occur, and less Ca is present in plasma. Similar effects of varying concentrations of dietary Ca and P on plasma Ca concentration were observed in 25-kg pigs [39]. In a similar experiment, 4 levels of dietary P and 5 levels of dietary Ca were fed to pigs from 25 to 50 kg, but dietary Ca only influenced the concentration of Ca in plasma when dietary P was deficient [3]. In the current experiment, the greatest variability in plasma Ca concentration was observed in the P-deficient diets; whereas, in diets with adequate dietary P, Ca concentrations were within a narrow range. The physiological range of serum Ca concentration is from 8 to 12 mg/dL [40]. Plasma Ca concentration is regulated by parathyroid hormone, vitamin D, and calcitonin [41]. Phosphorus concentration in plasma, however, is less tightly regulated than Ca concentrations [41] and data from this experiment confirm this hypothesis. Nevertheless, the observation that increased Ca addition to diets with the least and the greatest STTD P concentrations (0.16% and 0.50%, respectively) resulted in increased plasma P concentrations was unexpected and we do not have an explanation for this observation.

The process of Ca absorption from the intestine, which is important for maintaining Ca homeostasis [42], is carried out by transcellular as well as paracellular absorption of Ca [43, 44]. Transcellular absorption is the primary route under low dietary Ca conditions whereas paracellular transport is preferred if dietary Ca is at adequate or high levels [7, 9].

Transcellular absorption of Ca requires energy, Ca channels, and Ca-binding proteins [45]. Calcium is absorbed in the enterocyte through Ca channels such as TRPV6 located in the brush border membrane [8, 46]. In the cytosol, Ca is bound to Ca-binding proteins (calbindin-D9k), which move Ca towards the basolateral membrane [47, 48]. The release of Ca over the basolateral membrane is accomplished via plasma membrane Ca-ATPase activity, although Na^+^/Ca^2+^ exchange has also been identified [42]. This process is regulated by the active form of vitamin D (1, 25- dihydroxyvitamin D3), also known as calcitriol, which is activated in response to low plasma Ca concentration [8, 49, 50]. Calcitriol increases the absorption of Ca by upregulating genes related to Ca channels and transporters including TRPV6, Ca-binding proteins (e. g. S100G), and plasma membrane Ca-ATPase (e. g. ATP2B1) [51–54]. Results from this experiment demonstrated a decrease in the abundance of *S100G* and *TRPV6* in duodenal tissue as more Ca was included in the diet, which indicates reduced transcellular absorption of Ca in the duodenum of pigs fed high Ca diets. This can also be linked to the quadratic increase in plasma Ca concentration by increasing dietary Ca, which indicates that transcellular absorption is increased in pigs fed low Ca diets to elevate the concentration of Ca in plasma and is inhibited as the upper limit for plasma Ca is approached. However, dietary Ca appeared not to influence *ATP2B1* abundance. These observations are in agreement with data for Ca absorption in the jejunum of 50 kg pigs fed diets with increasing levels of Ca [6]. The lack of a response in *ATP2B1* abundance is not surprising because the influence of calcitriol on plasma membrane Ca-ATPase activity has not been consistently observed [42]. It appears that the regulation takes place at the luminal site and via the transport protein. This observation implies that transport out of the enterocyte to the interstitial space is not limiting for the Ca status of the pig.

Paracellular transport of Ca is a non-saturable passive process that mainly occurs in the jejunum and ileum [9]. The paracellular pathway takes place via the intercellular route between enterocytes that allows the movement of small molecules and ions, but this process needs to be regulated to maintain selective permeability [55]. The tight junctions are located in the apical region of the intercellular space and function as a semipermeable barrier to the passage of ions and molecules [56]. Claudins and occludin are integral membrane proteins that conform the tight junction structure and zonula occludens-1 is a peripheral membrane that binds the integral membrane proteins [56]. Paracellular absorption of Ca is, therefore, a result of high concentration of Ca in the lumen that generates an electrochemical gradient across the epithelium and influences the flux of Ca through the tight junctions [45]. This observation concurs with results obtained in this experiment that demonstrated that abundance of *OCLN* and *ZO1* in the duodenum and *CLDN1* and *ZO1* in the ileum was reduced as more Ca was included in the diet. Even though Ca absorption via the paracellular pathway is mainly performed in the jejunum and ileum, the present data indicate that passive absorption of Ca may start in the duodenum. A negative effect of high dietary Ca on the abundance of tight junction proteins was previously observed in the jejunum of young pigs [14]. This response indicates increased paracellular absorption of Ca by increasing dietary Ca and may imply a risk to the intestinal integrity if Ca is supplied above the requirement.

## Conclusions

If the concentration of P is deficient or at the estimated requirement in diets for 11- to 25-kg pigs, increasing concentrations of Ca are detrimental to growth performance. However, if P is above the requirement, the negative effect of increasing concentrations of dietary Ca is ameliorated, and increased dietary Ca may increase growth performance of pigs. The STTD Ca to STTD P ratio that maximizes bone ash is greater than the ratio that maximizes growth performance, indicating that pigs can utilize more Ca and P for bone tissue synthesis than for growth performance. The STTD Ca:STTD P ratio needed to maximize growth performance of 11- to 25-kg pigs is less than 1.40:1. Increasing dietary Ca increases plasma Ca concentration and decreases abundance of genes related to transcellular absorption and transport of Ca in the duodenum, but decreases abundance of tight junction proteins in the duodenum and ileum, which may result in increased paracellular absorption.

## Additional file

Additional file 1: **Table S1.** Gene-specific primer sets. (DOCX 17 kb)

## Abbreviations

ACTB: β-actin
ADF: Acid detergent fiber
ADFI: Average daily feed intake
ADG: Average daily gain
AEE: Acid hydrolyzed ether extract
ATP2B1: ATPase, Ca^2^^+^ transporting, plasma membrane-1
BW: Body weight
CLDN1: Claudin-1
CP: Crude protein
DCAD: Dietary cation-anion difference
DM: Dry matter
G:F: Gain:Feed
GAPDH: Glyceraldehyde 3-phosphate dehydrogenase
GE: Gross energy
HMBS: Hydroxymethylbilane synthase
NDF: Neutral detergent fiber
OCLN: Occludin
S100G: S100 calcium binding protein G
STTD: Standardized total tract digestible
TRPV6: Transient receptor potential cation channel, subfamily V, member 6
ZO1: Zonula occludens-1

## References

[1] NRC (2012). Nutrient requirements of swine. 11th rev.

[2] Stein HH, Merriman LA, González-Vega JC, Walk CL, Kühn I, Stein HH, Kidd MT, Rodehutscord M (2016). Establishing a digestible calcium requirement for pigs. Phytate destruction - consequences for precision animal nutrition.

[3] González-Vega JC, Walk CL, Murphy MR, Stein HH (2016). Requirement for digestible calcium by 25 to 50 kg pigs at different dietary concentrations of phosphorus as indicated by growth performance, bone ash concentration, and calcium and phosphorus balances. J Anim Sci.

[4] Lagos LV, Walk CL, Murphy MR, Stein HH (2019). Effects of dietary digestible calcium on growth performance and bone ash concentration in 50- to 85-kg growing pigs fed diets with different concentrations of digestible phosphorus. Anim Feed Sci Technol.

[5] Merriman LA, Walk CL, Murphy MR, Parsons CM, Stein HH (2017). Inclusion of excess dietary calcium in diets for 100- to 130-kg growing pigs reduces feed intake and daily gain if dietary phosphorus is at or below the requirement. J Anim Sci.

[6] González-Vega JC, Liu Y, McCann JC, Walk CL, Loor JJ, Stein HH (2016). Requirement for digestible calcium by eleven- to twenty-five–kilogram pigs as determined by growth performance, bone ash concentration, calcium and phosphorus balances, and expression of genes involved in transport of calcium in intestinal and kidney cells. J Anim Sci.

[7] Bronner F (2003). Mechanisms of intestinal calcium absorption. J Cell Biochem.

[8] Bouillon R, Cromphaut SV, Carmeliet G (2003). Intestinal calcium absorption: molecular vitamin D mediated mechanisms. J Cell Biochem.

[9] Pérez AV, Picotto G, Carpentieri AR, Rivoira MA, Peralta López ME, Tolosa de Talamoni NG (2008). Minireview on regulation of intestinal calcium absorption. Emphasis on molecular mechanisms of transcellular pathway. Digestion..

[10] Kim BG, Lindemann MD (2007). A new spreadsheet method for the experimental animal allotment. J Anim Sci.

[11] AOAC Int. Official methods of analysis of AOAC Int. 18th ed. Rev.2. Gaithersburg, MD. USA. Assoc. Off. Anal. Chem; 2007.

[12] AOAC Int (2006). Official methods of analysis of AOAC int.

[13] Vailati-Riboni M, Meier S, Priest NV, Burke CR, Kay JK, McDougall S (2015). Adipose and liver gene expression profiles in response to treatment with a nonsteroidal antiinflammatory drug after calving in grazing dairy cows. J Dairy Sci.

[14] Metzler-Zebeli BU, Mann E, Ertl R, Schmitz-Esser S, Wagner M, Klein D (2015). Dietary calcium concentration and cereals differentially affect mineral balance and tight junction proteins expression in jejunum of weaned pigs. Br J Nutr.

[15] Vigors S, Sweeney T, O'Shea CJ, Browne JA, O'Doherty JV (2014). Improvements in growth performance, bone mineral status and nutrient digestibility in pigs following the dietary inclusion of phytase are accompanied by modifications in intestinal nutrient transporter gene expression. Br J Nutr.

[16] Tran G, Sauvant D, Ponter A, Sauvant D, Perez J-M, Tran G (2004). Chemical data and nutritional value. Tables of composition and nutritional value of feed materials: pigs, poultry, cattle, sheep, goats, rabbits, horses and fish.

[17] Sherrod PH. Nonlinear regression analysis program (NLREG) version 6.5 (advanced). Nashville, TN. Philip H. Sherrod. 2008.

[18] Khuri AI, Cornell JA (1996). Response surfaces: designs and analyses.

[19] Lindemann MD, Kim BG (2007). Technical note: a model to estimate individual feed intake of swine in group feeding. J Anim Sci.

[20] de Blas C, Mateos GG, García-Rebollar P (2010). Tablas FEDNA de composición y valor nutritivo de alimentos para la fabricación de piensos compuestos. (In spanish). 3rd. ed.

[21] Rostagno HS, Albino LFT, Donzele JL, Gomes PC, de Oliveira RF, Lopes DC (2011). Brazilian tables for poultry and swine. Composition of feedstuffs and nutritional requirements. 3rd ed.

[22] Sauvant D, Perez JM, Tran G (2004). Tables of composition and nutritional value of feed materials: pigs, poultry, cattle, sheep, goats, rabbits, horses and fish.

[23] Stein HH, Lagos LV, Casas GA (2016). Nutritional value of feed ingredients of plant origin fed to pigs. Anim Feed Sci Technol.

[24] Dersjant-Li Y, Schulze H, Schrama JW, Verreth JA, Verstegen MWA (2001). Feed intake, growth, digestibility of dry matter and nitrogen in young pigs as affected by dietary cation–anion difference and supplementation of xylanase. J Anim Physiol Anim Nutr.

[25] Vier C. M., Wu F., Dritz S. S., Tokach M. D., Goncalves M. A. D., Orlando U. A. D., Woodworth J. C., Goodband R. D., DeRouchey J. M. (2017). 119 Standardized total tract digestible phosphorus requirement of 11- to 25-kg pigs. Journal of Animal Science.

[26] Box G. E. P., Wilson K. B. (1951). On the Experimental Attainment of Optimum Conditions. Journal of the Royal Statistical Society: Series B (Methodological).

[27] Steel RGD, Torrie JH, Dickey DA (1996). Principles and procedures of statistics: a biometrical approach.

[28] Xie M, Wang SX, Hou SS, Huang W (2009). Interaction between dietary calcium and non-phytate phosphorus on growth performance and bone ash in early white Pekin ducklings. Anim Feed Sci Technol.

[29] Akter MM, Graham H, Iji PA (2017). Influence of different levels of calcium, non-phytate phosphorus and phytase on apparent metabolizable energy, nutrient utilization, plasma mineral concentration and digestive enzyme activities of broiler chickens. J Appl Anim Res.

[30] Wu F, Tokach MD, Dritz SS, Woodworth JC, DeRouchey JM, Goodband RD (2018). Effects of dietary calcium to phosphorus ratio and addition of phytase on growth performance of nursery pigs. J Anim Sci.

[31] Brink EJ, Beynen AC, Dekker PR, van Beresteijn ECH, van der Meer R (1992). Interaction of calcium and phosphate decreases ileal magnesium solubility and apparent magnesium absorption in rats. J Nutr.

[32] Stein HH, Adeola O, Cromwell GL, Kim SW, Mahan DC, Miller PS (2011). Concentration of dietary calcium supplied by calcium carbonate does not affect the apparent total tract digestibility of calcium, but decreases digestibility of phosphorus by growing pigs. J Anim Sci.

[33] Walk CL, Addo-Chidie EK, Bedford MR, Adeola O (2012). Evaluation of a highly soluble calcium source and phytase in the diets of broiler chickens. Poult Sci.

[34] González-Vega JC, Walk CL, Liu Y, Stein HH (2014). The site of net absorption of ca from the intestinal tract of growing pigs and effect of phytic acid, ca level and ca source on ca digestibility. Arch Anim Nutr.

[35] Mutucumarana RK, Ravindran V, Ravindran G, Cowieson AJ (2014). Influence of dietary calcium concentration on the digestion of nutrients along the intestinal tract of broiler chickens. J Poult Sci.

[36] Crenshaw TD, Danielson JR, Hoffman LE, Schneider DK (2009). Femurs are more accurate than fibulas as predictors of whole body bone mineral content in growing pigs. J Anim Sci.

[37] Crenshaw TD, Lewis AJ, Southern LL (2001). Calcium, phosphorus, vitamin D, and vitamin K in swine nutrition. Swine nutrition.

[38] Suttle NF (2010). Mineral nutrition of livestock.

[39] Nicodemo ML, Scott D, Buchan W, Duncan A, Robins SP (1998). Effects of variations in dietary calcium and phosphorus supply on plasma and bone osteocalcin concentrations and bone mineralization in growing pigs. Exp Physiol.

[40] Amundson Laura A., Hernandez Laura L., Crenshaw Thomas D. (2017). Serum and tissue 25-OH vitamin D3 concentrations do not predict bone abnormalities and molecular markers of vitamin D metabolism in the hypovitaminosis D kyphotic pig model. British Journal of Nutrition.

[41] Veum TL, Vitti DMSS, Kebreab E (2010). Phosphorus and calcium nutrition and metabolism. Phosphorus and calcium utilization and requirements in farm animals.

[42] Schröder B, Breves G (2007). Mechanisms and regulation of calcium absorption from the gastrointestinal tract in pigs and ruminants: comparative aspects with special emphasis on hypocalcemia in dairy cows. Anim Health Res Rev.

[43] Hurwitz S (1996). Homeostatic control of plasma calcium concentration. Crit Rev Biochem Mol Biol.

[44] Taylor JG, Bushinsky DA (2009). Calcium and phosphorus homeostasis. Blood Purif.

[45] Gropper SS, Smith JL (2013). Advanced nutrition and human metabolism.

[46] van de Graaf SFJ, Boullart I, Hoenderop JGJ, Bindels RJM. Regulation of the epithelial Ca^2+^ channels TRPV5 and TRPV6 by 1α,25-dihydroxy vitamin D_3_ and dietary Ca^2+^. J Steroid Biochem Mol Biol. 2004;89(90):303–8. 10.1016/j.jsbmb.2004.03.029 15225790

[47] Kaune R (1996). Mechanisms of intestinal calcium absorption and availability of dietary calcium in pigs. Dtsch Tierarztl Wochenschr.

[48] Schwaller B. (2010). Cytosolic Ca2+ Buffers. Cold Spring Harbor Perspectives in Biology.

[49] Eklou-Kalonji E, Zerath E, Colin C, Lacroix C, Holy X, Denis I (1999). Calcium-regulating hormones, bone mineral content, breaking load and trabecular remodeling are altered in growing pigs fed calcium-deficient diets. J Nutr.

[50] Fleet JC, Schoch RD (2010). Molecular mechanisms for regulation of intestinal calcium absorption by vitamin D and other factors. Crit Rev Clin Lab Sci.

[51] Cai Q, Chandler JS, Wasserman RH, Kumar R, Penniston JT (1993). Vitamin D and adaptation to dietary calcium and phosphate deficiencies increase intestinal plasma membrane calcium pump gene expression. Proc Natl Acad Sci U S A.

[52] Ko S-H, Lee G-S, Vo TTB, Jung E-M, Choi K-C, Cheung K-W (2009). Dietary calcium and 1,25-dihydroxyvitamin D_3_ regulate transcription of calcium transporter genes in calbindin-D9k knockout mice. J Reprod Dev.

[53] Armbrecht HJ, Boltz MA, Bruns MEH (2003). Effect of age and dietary calcium on intestinal calbindin D-9k expression in the rat. Arch Biochem Biophys.

[54] Kutuzova Galina D., DeLuca Hector F. (2004). Gene expression profiles in rat intestine identify pathways for 1,25-dihydroxyvitamin D3 stimulated calcium absorption and clarify its immunomodulatory properties. Archives of Biochemistry and Biophysics.

[55] Hoenderop JG, Nilius B, Bindels RJ (2005). Calcium absorption across epithelia. Physiol Rev.

[56] Chiba H, Osanai M, Murata M, Kojima T, Sawada N (2008). Transmembrane proteins of tight junctions. Biochim Biophys Acta.

