# Supplementary material for: Influence of the concentration of dietary digestible calcium on growth performance, bone mineralization, plasma calcium, and abundance of genes involved in intestinal absorption of calcium in pigs from 11 to 22 kg fed diets with different concentrations of digestible phosphorus
Source: J Anim Sci Biotechnol. 2019 May 28;10:47. doi: 10.1186/s40104-019-0349-2 (PMC6537374; doi:10.1186/s40104-019-0349-2)
Supplement: Supplementary file 1 — Table S1. Gene-specific primer sets. (DOCX 17 kb) [file 40104_2019_349_MOESM1_ESM.docx]

Table S1 Gene-specific primer sets

| Target^1^ | Forward | Reverse | Length, bp | Source |
| --- | --- | --- | --- | --- |
| Transcellular absorption and transport of Ca | |  |  |  |
| *ATP2B1* | 5’-GGGCGGGCAGGTCATT-3’ | 5’-CCGCCGGGAGAAGATCA-3’ | 86 | Vigors et al. [1] |
| *TRPV6* | 5’-CCAGACAGAGGACCCTAACAAG-3’ | 5’GTGAGAAACAGCTCAAAGGTGCTA-3’ | 82 | Vigors et al. [1] |
| *S100G* | 5’-CGCAACAGTCCCATTTAAGGA-3’ | 5’-TCAGCAGAGACATGGGTGGTT-3’ | 72 | Vigors et al. [1] |
| Paracellular absorption of Ca | |  |  |  |
| *OCLN* | 5’-TCCTGGGTGTGATGGTGTTC-3’ | 5’-CGTAGAGTCCAGTCACCGCA-3’ | 145 | Hu et al. [2] |
| *ZO1* | 5’AAGCCCTAAGTTCAATCACAATCT-3’ | 5’-ATCAAACTCAGGAGGCGGC-3’ | 130 | Hu et al. [2] |
| *CLDN1* | 5’-AGAAGATGCGGATGGCTGTC-3’ | 5’-CCCAGAAGGCAGAGAGAAGC-3’ | 193 | Hu et al. [2] |
| Internal control genes | |  |  |  |
| *β-ACTIN* | 5’-GGATGCAGAAGGAGATCACG-3’ | 5’-ATCTGCTGGAAGGTGGACAG-3’ | 150 | Lackeyram et al. [3] |
| *GAPDH* | 5’-TTCGTCAAGCTCATTTCCTGGTA-3’ | 5’-TCCTCGCGTGCTCTTGCT-3’ | 130 | Vigors et al. [1] |
| *HMBS* | 5’-CTGAACAAAGGTGCCAAGAACA-3’ | 5’-GCCCCGCAGACCAGTTAGT-3’ | 74 | Vigors et al. [1] |

^1^*ATP2B1*= ATPase, Ca^2+^ transporting, plasma membrane-1; *TRPV6*= transient receptor potential cation channel, subfamily V, member 6; *S100G*= S100 calcium binding protein G; *OCLN*= Occludin; *ZO1* = Zonula occludens-1; *CLDN1*= Claudin-1; *ACTB*= β-actin; *GAPDH*= glyceraldehyde 3-phosphate dehydrogenase; *HMBS*= hydroxymethylbilane synthase.

References

[1] Vigors S, Sweeney T, O'Shea CJ, Browne JA, O'Doherty JV. Improvements in growth performance, bone mineral status and nutrient digestibility in pigs following the dietary inclusion of phytase are accompanied by modifications in intestinal nutrient transporter gene expression. Br J Nutr. 2014;112:688–97.

[2] Hu CH, Xiao K, Luan ZS, Song J. Early weaning increases intestinal permeability, alters expression of cytokine and tight junction proteins, and activates mitogen-activated protein kinases in pigs. J Anim Sci. 2013;91:1094–101.

[3]  Lackeyram D, Yang C, Archbold T, Swanson KC, Fan MZ. Early weaning reduces small intestinal alkaline phosphatase expression in pigs. J Nutr. 2010;140:461–8.
